# Depth Sensitivity and Source-Detector Separations for Near Infrared Spectroscopy Based on the Colin27 Brain Template

**DOI:** 10.1371/journal.pone.0066319

**Published:** 2013-08-01

**Authors:** Gary E. Strangman, Zhi Li, Quan Zhang

**Affiliations:** Neural Systems Group, Massachusetts General Hospital/Harvard Medical School, Charlestown, Massachusetts, United States of America; Institute of Psychology, Chinese Academy of Sciences, China

## Abstract

Understanding the spatial and depth sensitivity of non-invasive near-infrared spectroscopy (NIRS) measurements to brain tissue–i.e., near-infrared neuromonitoring (NIN) – is essential for designing experiments as well as interpreting research findings. However, a thorough characterization of such sensitivity in realistic head models has remained unavailable. In this study, we conducted 3,555 Monte Carlo (MC) simulations to densely cover the scalp of a well-characterized, adult male template brain (Colin27). We sought to evaluate: (i) the spatial sensitivity profile of NIRS to brain tissue as a function of source-detector separation, (ii) the NIRS sensitivity to brain tissue as a function of depth in this realistic and complex head model, and (iii) the effect of NIRS instrument sensitivity on detecting brain activation. We found that increasing the source-detector (SD) separation from 20 to 65 mm provides monotonic increases in sensitivity to brain tissue. For every 10 mm increase in SD separation (up to ∼45 mm), sensitivity to gray matter increased an additional 4%. Our analyses also demonstrate that sensitivity in depth (S) decreases exponentially, with a “rule-of-thumb” formula S = 0.75*0.85^depth^. Thus, while the depth sensitivity of NIRS is not strictly limited, NIN signals in adult humans are strongly biased towards the outermost 10–15 mm of intracranial space. These general results, along with the detailed quantitation of sensitivity estimates around the head, can provide detailed guidance for interpreting the likely sources of NIRS signals, as well as help NIRS investigators design and plan better NIRS experiments, head probes and instruments.

## Introduction

Near-infrared spectroscopy (NIRS) and diffuse optical imaging (DOI) have been successfully used for non-invasive assessment of brain hemodynamics for over three decades [1], [2], [3]. Properly interpreting any type of non-invasive measurement – including NIRS and DOI – requires detailed knowledge about the measurement's spatial sensitivity profile. In general, the question is what tissues are being probed by a given measurement? A more specific question commonly asked is, what is the depth penetration of NIN? Addressing these questions is particularly difficult because light propagation through scattering media with heterogeneous structure (such as the head) is inherently complex, and because the governing mathematical models of this process – the radiative transport equation and its diffusion approximation – are difficult to solve analytically for any but the most trivial of tissue geometries [4], [5], [6], [7]. It is even more difficult to empirically measure light fluence within tissue, as this would require the placement of omnidirectional light detectors inside a probed medium.

For simple tissue geometries, such as optically homogeneous tissues with infinite, semi-infinite or slab boundary conditions, analytical solutions to the diffusion equation have been developed [8], [9], [10], [11], [12], [13]. However, even modestly more complex geometries, and especially the irregular boundaries and undulating layers found in brain tissue, have no analytical solutions. As a result, far less is understood about the photon distribution through the head and, consequently, NIRS sensitivity to brain tissue.

In the absence of direct observation or analytical solutions, questions about sensitivity and penetration depth in complex tissues must instead rely on numerical approaches. There are two general categories: (1) approaches based on finite element (FE) and finite difference (FD) analysis [14], [15], or (2) Monte Carlo simulations of photon propagation through the tissue in question [16]. The appeal of FE and FD techniques is that they can handle arbitrary tissue type boundary conditions yet require considerably less computation time than Monte Carlo methods [15]. A shortcoming of FE/FD techniques is that they need to assume an analytical form for the photon migration through tissue. Typically (though not always, e.g. [17]), the diffusion approximation is assumed. While the diffusion approximation is generally well supported, it is difficult to test in detail the required assumptions, particularly for complex tissue boundary conditions such as the undulating layers inside a human head.

The other numerical technique typically employed is Monte Carlo (MC) simulation [16], [18], [19], [20]. This technique proceeds as follows: (1) select a three-dimensional tissue geometry and divide it into voxels of different tissue types, (2) assign scattering and absorption optical properties to each voxel based on tissue type, (3) select a point on the surface of this geometry and “inject” a photon at that point, (4) propagate that photon through the tissue by allowing it to probabilistically scatter and be absorbed as it travels, and (5) repeat steps 3–4 many thousands or millions of times and accumulate the resulting photon weights (or, fluences) along with the cumulative distance traveled through each tissue type. The Monte Carlo approach is unfortunately quite computationally intensive. However, it provides the most accurate estimate of photon propagation through irregular boundary conditions and heterogeneous media such as the human brain. Importantly, it does not need to assume an analytical form for photon propagation, and hence is not biased by diffusion or other approximations [21], [22].

A number of groups have used Monte Carlo methods to investigate NIRS sensitivity in layered tissues. Most such studies, however, have only considered relatively simple geometries such as layered slabs [23], [24], [25], [26], [27], cylinders [28], [29], [30], or concentric spheres [31]. In general, these studies confirm general expectations based on diffusion theory: (1) depth sensitivity increases as source-detector separation increases [31], [32], (2) overlying tissue layers interfere with the measurement of deeper layers [30], [33], [34], (3) sensitivity to deeper layers decreases with increasing thickness of overlying tissue layers [24], [25], (4) depth sensitivity is affected by the angle at which incident photons contact the surface [28], and (5) there is an improvement of NIRS sensitivity to deeper tissue layers when modeling scattering elements are embedded in cerebrospinal fluid (CSF) layers [26]. These studies have provided very useful guides, but the use of simple geometries means that the results cannot be assumed to apply to brain geometries, at least not quantitatively.

An important set of studies focused more specifically on the role that the relatively clear CSF layer may play in NIN measurements [23], [24], [25], [26], [29], [35], [36], [37], [38]. For example, at least one study suggested that CSF could distort the sensitivity profile, both broadening it and reducing the sensitivity to brain with increasing CSF thicknesses from 0.5 to 5 mm [25]. The CSF effect also appears strongly modulated by the precise amount of scattering within the CSF layer itself [24], [26], [35], [36]. These CSF-related effects appear to be substantial relative to other tissue distribution issues [38].

Six studies have examined realistic human brain models based on structural MRI scans [32], [34], [35], [38], [39], [40]. All of these studies employed a handful of Monte Carlo simulations, focusing on a particular location of the head. NIRS sensitivity to functional brain activation was concluded to be “high” [34], at least for source-detector (SD) separations in the 30–35 mm range [32], [39]. In an MC study on two separate MRI-based head models of pre-term infants, using 1 source plus 20 detectors around the head, differences in tissue distributions were found to affect measured NIRS signals, but these effects were modest compared to the effects generated by the relatively clear CSF [38]. Another study examined whether the CSF effect was sufficient to invalidate the use of the diffusion approximation for computing photon propagation through head tissue [40]. Using 1 source and 25 linearly aligned detectors in an MRI-based adult head phantom, sensitivity to the brain reached approximately 11% of the sensitivity profile at source-detector (SD) separations of 40 mm. This sensitivity was slightly reduced in the face of larger scattering coefficients, but the overall conclusion was that a relatively scattering CSF layer (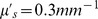) was unlikely to generate more than 20% error in sensitivity estimates when using the diffusion approximation.

Most recently, Mansouri and colleagues combined an MRI-based head model and a highly resource-intensive MC approach to assess the sensitivity of NIRS to brain tissue over one location in the left frontal pole [35]. Using four simulations, and examining source-detector (SD) separations of 20, 33 and 40 mm, they found sensitivity to brain tissue ranged from approximately 1% of the total NIRS sensitivity (99% coming from scalp and skull) at a 20 mm SD separation, to 6–9% of the total NIRS sensitivity at 40 mm. The maximum sensitivity again depended on the amount of scattering in the CSF layer, with higher scattering (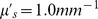) leading to greater brain sensitivity as found by Custo and colleagues.

The above findings–particularly when coupled to the known anatomical variability for scalp, skull, CSF, gray and white matter within and between people and brain regions – suggest that small numbers of MC runs are likely to be biased by regional details in tissue types and layer thicknesses. The available literature, however, leaves a notable gap in terms of a more comprehensive analysis of photon propagation across realistic head geometries. First, the current maximum of less than 10 Monte Carlo runs in a single head model is insufficient for comprehensively and robustly evaluating NIRS sensitivity to complex tissues such as the brain, much less providing variability measures of such sensitivity. Second, there has been limited quantification of NIRS sensitivity to brain versus non-brain tissue compartments as a function of source-detector separation, or in relation to instrument sensitivity. And third, NIRS sensitivity as a function of depth remains particularly poorly understood for complex head geometries, yet this is key information when trying to interpret NIN data. Here we address each of these issues using over 3,555 Monte Carlo simulations in a detailed, five-layer model of the standard Colin27 human head template.

## Materials and Methods

### Brain Tissue Model

**Figure 1:**
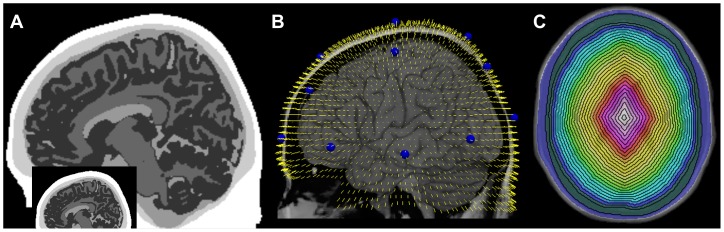
Anatomy used for Monte Carlo simulations and processing. (A) Sagittal section (MNIx = −9 mm) through the segmented Colin27 head. Shades from white to dark gray are: scalp, skull, cerebrospinal fluid, white and gray matter, respectively. The inset shows the original, pre-eroded scalp on the same slice. (B) Location and orientation of the 3,555 photon injection points around the Colin27 scalp used for the Monte Carlo simulations. The injection vectors (yellow) are shown in relation to the scalp profile and underlying cortical surface (rendered brain). The nineteen standard locations in the International 10–20 System are highlighted in blue. (C) Colorized shells representing the masks used for depth sensitivity analysis (blue = scalp, green = skull, dark blue to pink = twenty-one ∼2.8 mm thick shells).

For a well-characterized starting point, we used the 1×1×1 mm Colin27 template brain [41], as distributed with FSL v4.1 [42]. To this scan, we applied the default SPM8 segmentation process [43] which generated three tissue type probability images: gray matter, white matter and cerebrospinal fluid (CSF). Each probability map was slightly smoothed (0.75 mm FWHM Gaussian kernel) and then intracranial voxels were classified as gray matter, white matter or CSF. A voxel was classified as gray matter if it had the highest probability of being gray matter and this probability was greater than 20%. White matter was treated similarly. An intracranial voxel was then classified as CSF if it had a higher probability than both gray and white matter. Holes were filled with the modal nearest-neighbor tissue type (less than 0.02% of all voxels). The original Colin27 template was also passed through FSL's brain extraction tool [44] to generate three masks: scalp, skull, and intracranial tissue. The scalp and skull masks were merged with the above CSF, gray and white matter segmented volume to generate a whole head model with 5 tissue types: scalp, skull, CSF, gray matter, and white matter. Upon measurement, the mean scalp thickness was found to be 11.4±3.0 mm (for MNIz>−20 mm; Figure 1A-inset), which is substantially at odds with reported values [45], [46], [47], [48]. This could arise due to various factors related to the MRI acquisition and multi-scan averaging. Hence, we eroded the scalp layer by 3 single-voxel steps using Freesurfer's mri_binarize tool, resulting in a mean scalp thickness of 6.9±3.6 mm which is more in line with prior findings. We proceeded with the 5-layer model with the eroded scalp (Figure 1A).

For the Monte Carlo simulations, optical properties were assigned to the five tissue types, per Table 1. These values represent the mean of published optical properties across four typically-used NIRS wavelengths: 690, 750, 780 and 830nm [23], [49], [50]. For all tissue types, the tissue anisotropy parameter (g) was set to 0.01–representing predominantly forward-scattered light – and the tissue index of refraction (n) was set to 1.

**Table 1:** Optical properties for scalp, skull, CSF, gray and white matter used for all Monte Carlo simulations.

Tissue Type	*μ_a_*(*mm^−^* ^1^)	*μ_s_*(*mm^−^* ^1^)
Gray matter	0.019500	1.10
White matter	0.016900	1.35
CSF	0.002500	0.01
Skull	0.011925	0.92
Scalp	0.017275	0.72

### Monte Carlo Injection Points

Some 5,000 points were initially spaced 5 mm apart on a 95 mm radius sphere (the approximate radius of the Colin27 head). The sphere was centered within the Colin27 volume, and the sphere points were moved radially toward or away from this center voxel until they reached the surface of the Colin27 scalp. This location list was then pruned to remove all points below the cerebellum (MNIz<−60 mm) as well as points that were both below the eyes (MINz<−35 mm) and anterior to the temporal pole (MNIy>+35 mm). The resulting 3,555 points were spaced 3–6 mm apart and covered all scalp regions overlying brain tissue (Figure 1B). These points constituted the source locations and directions for photon injection during Monte Carlo simulations.

### Monte Carlo Simulations

We employed a three-dimensional Monte Carlo (MC) method based on the tMCimg code described by Boas et al. [51], with the general approach being described by Wang [52]. In brief, the initial position and direction of a photon are defined as coming from a point source with an initial survival weight 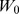 set to 1. A scattering length *L* is probabilistically calculated from an exponential distribution, and the photon is moved through tissue by this length. The photon's weight, is incrementally decreased by a factor of 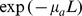 due to absorption, where 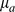
is the absorption coefficient of the tissue and 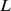
is the length traveled by the photon. A scattering angle is then calculated using the probability distribution given by the Henyey-Greenstein phase function, and a new scattering length is determined from an exponential distribution. The photon is moved the new distance in the updated direction defined by the scattering angle. This process continued until the photon exited the medium or traveled longer than 10ns, since the probability of photon detection in perfused tissue after such a period of time is extremely small. When the photon reaches a boundary, the probability of an internal reflection is calculated from Fresnel's formula. If a reflection occurs, the photon is reflected back into the medium and propagation continues with a new distance. Otherwise, the migration of this photon is terminated and a new photon is launched. The output of a given Monte Carlo simulation included the photon fluence within the medium: an accumulation of all photon weights within each voxel in the tissue, also known as the 2-point Green's function. In addition, for each detector position the MC simulation stores in a history file: (1) the number of photons exiting within a 3 mm diameter of that detector location, and (2) every photon's path length traveled through each tissue type that reached that detector. For each of the 3,555 simulations in our study, 100 million (i.e., 10^8^) input photons were injected at the source location. Individual MC simulations required 4–5 hours of computation time and 1–2 GB of storage (uncompressed).

### Sensitivity Map Computation

**Figure 2:**
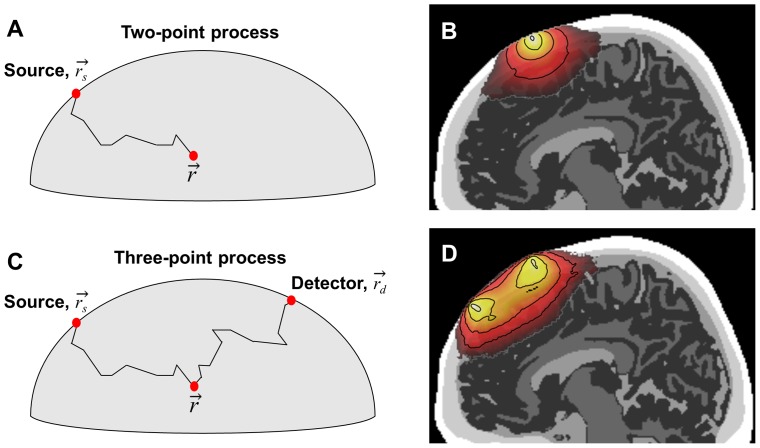
Photon propagation through scattering tissue. (A) Representation of a single photon moving through tissue, from the source, to an arbitrary point inside the medium. Accumulation of photon weights during this process is the basis of a 2-point Green's function. (B) Example 2-point Green's function, with colors representing the intensity of light reaching any given point in the tissue (truncated after a 5 order-of-magnitude reduction in intensity from peak). (C) Representation of a single photon traveling from the source, to a point in the medium, and on to a detector; the basis of a 3-point sensitivity function. (D) Example 3-point sensitivity function generated from two MC simulations (one for the source, one for the detector) spaced 30 mm apart.

Unlike x-rays which typically pass straight through biological tissue, near-infrared photons scatter significantly as they travel through tissue. The spatial probability distribution of photons entering tissue at a source location, scattering through the tissue, and being emitted at a particular detector location, defines the spatial sensitivity profile (i.e., the “probed tissue”) for that source-detector (SD) pair. In linear DOI image reconstruction, this spatial probability distribution is represented by a 3-point Green's function (see Eqn 1), which can be computed from two separate 2-point Green's functions (i.e., 2 separate MC results), as described below (cf. Figure 2).

The probability of a photon traveling from a point source, 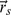, to any particular voxel inside the head, 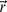, is represented by a single 2-point Green's function (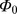
in Eqn 1; see also Figure 2A,B). The probability of a photon traveling from a point source, to any particular voxel inside the head and then arriving a particular detector location, 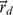, is represented by a 3-point Green's function [53], ***W*** (see Figure 2C,D). The 3-point Green's function is formally given by:
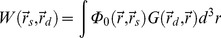
(1)where 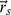
and 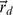
are the locations of the source and detector, respectively, and 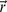 is a position inside the head. 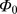 is the fluence provided by the MC 2-point file for the source point (e.g. Figure 2B), and the function *G* is provided by a separate MC 2-point file for the detector point. For continuous-wave NIRS measurements, the 3-point function (e.g., Figure 2D) can be generated by simply multiplying the 2-point function obtained from the source location (e.g., Figure 2B) by the 2-point function from the detector location, voxel by voxel. [For energy conservation, the data stored in each tMCimg 2-point file is first normalized according to Eqn. (1) in [51].] Once computed, the 3-point function *W* represents the sensitivity of the optical measurement for detecting changes at any point inside the medium, or a “spatial sensitivity profile”, from that SD pair (Figure 2D) – typically described as roughly banana-shaped. We will refer to the sensitivity function *W* the “3- point sensitivity function”.

### Spatial Sensitivity Analyses

To estimate NIRS sensitivity to our five different tissue types within the head (e.g., Figure 1A), a given 3-point sensitivity map was partitioned and accumulated. The total fluence change to a perturbation in brain tissue, 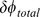, can be partitioned various ways based on the 3-point maps. For example, one option is to partition the total fluence change into two components: brain fluence and non-brain fluence:
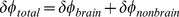
(2)


If we assume the absorption change in brain tissue sampled by that SD pair is uniform (and similarly for the non-brain tissue), these can be represented by 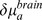 and 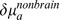 respectively. The total fluence change can then be written in terms of the 3-point functions ***W***, partitioned into brain and non-brain components:
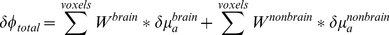
(3)


Since sensitivity is defined as the change in optical fluence divided by change in *μ_a_* (assuming scattering is constant), the sensitivity to brain tissue and non-brain tissue can be derived by taking the partial derivative of the above equation with respect to 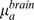 and 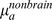, respectively:
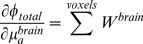
(4)

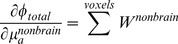
(5)


The sensitivity to one partition (e.g., brain tissue) can therefore be estimated by summing over all voxels in the 3-point weighting function that comprise that partition, 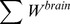. This concept generalizes from a brain/non-brain partition to any other partitioning of the head. For example, one could separately sum over all gray matter voxels to compute the cumulative sensitivity to gray matter, or sum over white matter voxels to compute the sensitivity to white matter. One could also use geometric partition partitions of the head, such as concentric “depth” shells described further below.

#### Regional and global sensitivity analysis

To assess regional NIRS sensitivity around the head, we performed sensitivity analyses at the 19 standard points of the International 10–20 System in MNI coordinate space [54]. For each of the 19 locations, we first identified every pair of points from our 3,555 MCs that were separated by less than 60 mm and whose midpoint lay within 10 mm of the given 10–20 location. We then generated a 3-point map for each pair identified, and summed the 3-point weights for each of the five tissue types to quantify the probability of photons passing through each tissue type. This partitioning and sensitivity computation was repeated for each pair in the list, retaining SD separation information. Sensitivity and variability was quantified by averaging results surrounding a given 10–20 location, in 5 mm bins. For example, to estimate sensitivity to gray matter given a 30 mm separation, we averaged together the gray matter partition sums for all SD pairs that were 27.5–32.5 mm apart and also centered on a point within 10 mm of the 10–20 location of interest. This entire process was repeated for each SD separation considered. Depending on the separation, anywhere from 30–100 such SD pairs contributed to the average.

The result for a given 10–20 location was an average curve representing the sensitivity and variability of a NIRS measurement to each tissue type as a function of SD separation, centered on the target location. The entire process was then repeated at each of the nineteen 10–20 locations to quantify sensitivity and variability as a function of position on the head. To estimate the mean global sensitivity to the five tissue types, we averaged the curves from the 19 standard locations of the 10–20 system. In this analysis, we dropped the error associated with individual SD-pairs surrounding individual locations to highlight the variability across 10–20 location.

In practice, intensity contours such as those shown in Figure 2 are linearly related to the measurement of signal change. Optical property changes occurring within the farthest contours from the source and detector will produce smaller signal changes. Hence, a more sensitive or lower-noise instrument will be required to detect signals arising from those regions. The farthest contour to which an instrument is sensitive, therefore, is characterized by the NIRS instrument's noise characteristics, sensitivity and dynamic range. In our study, the entire sensitivity analysis process was performed both before and after thresholding the 3-point functions at 5, 4, 3 or 2 orders of magnitude in sensitivity loss from peak (i.e., masking at the 5th, 4th, 3rd or 2nd contour line in Figure 2). This simulates instruments with progressively lower sensitivity, higher noise, and/or a more restricted dynamic range.

#### Depth sensitivity

To assess NIN depth sensitivity, the intracranial mask generated by FSL was eroded in three dimensions 21 times via the mri_binarize tool (FreeSurfer, v5.1), in two-voxel steps. Erosion in a voxellated space depends upon the surface curvature, but the mean erosion step was 2.8 mm. Successive mask-pairs were then subtracted to generate a series of twenty-one “shells”, each on average 2.8 mm thick, beginning at the inner edge of the skull and continuing toward the center of the brain. These were then combined with the scalp and skull masks to provide a separate head segmentation with 23 complete, non-overlapping shells (colors in Figure 1C). As with the regional sensitivity analysis, the same groups of 3-point sensitivity maps surrounding the nineteen 10–20 locations were partitioned using each of these 23 shells (i.e., ignoring the 3 intracranial tissue types). Sensitivity weights were summed to independently quantify cumulative photon fluence within each layer in depth, irrespective of the five tissue types used in the MC simulations themselves.

## Results

### Spatial Sensitivity for Near-Infrared Neuroimaging

**Figure 3:**
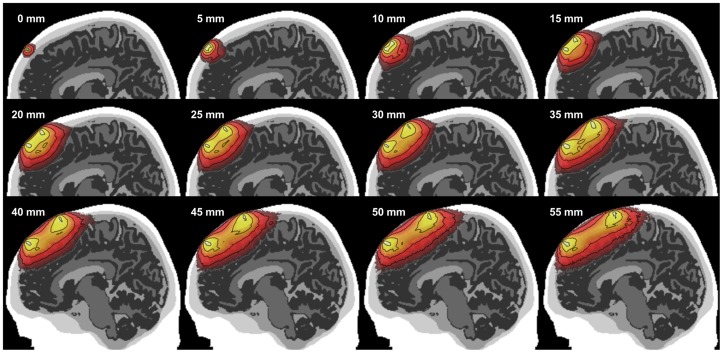
Photon sensitivity profile at a broad range of source-detector separations. Contours are drawn for each order of magnitude loss in sensitivity from peak and are truncated after 5 orders of magnitude.

A series of 3-point spatial sensitivity functions, ***W***, are shown in Figure 3 in a sagittal slice covering a wide range of SD separations. Contour lines appear at each order of magnitude decrease in the photon sensitivity profile from the peak voxel, and are truncated in each figure after decaying 5 orders-of-magnitude. This represents the approximate limits of a sensitive, low noise, and well-optimized NIRS measurement; less sensitive devices or poorly optimized dynamic range may only provide information up through the first three or four contour lines.

Consistent with previous findings, as separations increase from 0 to 55 mm, the NIRS “banana” grows and extends deeper into the brain. At a separation of 55 mm, the overlay shown intersects with roughly the outer 17 mm of brain tissue. Careful visual inspection of these examples also reveals that the relatively non-scattering CSF layer distorted the normally smooth ovoid shape of the NIRS banana that is found in a homogeneous medium [25]. Such distortion is particularly noticeable when the CSF layer is more than 1–2 millimeters thick and the CSF layer is near the edge of the sensitivity profile (see the 5 and 10 mm separations in Figure 3). These examples also suggest there is at least limited sensitivity to gray matter even at SD separations less than 20 mm – separations that are typically assumed to provide zero brain sensitivity in an adult human head.

### Sensitivity as a Function of Source-Detector Separation

**Figure 4:**
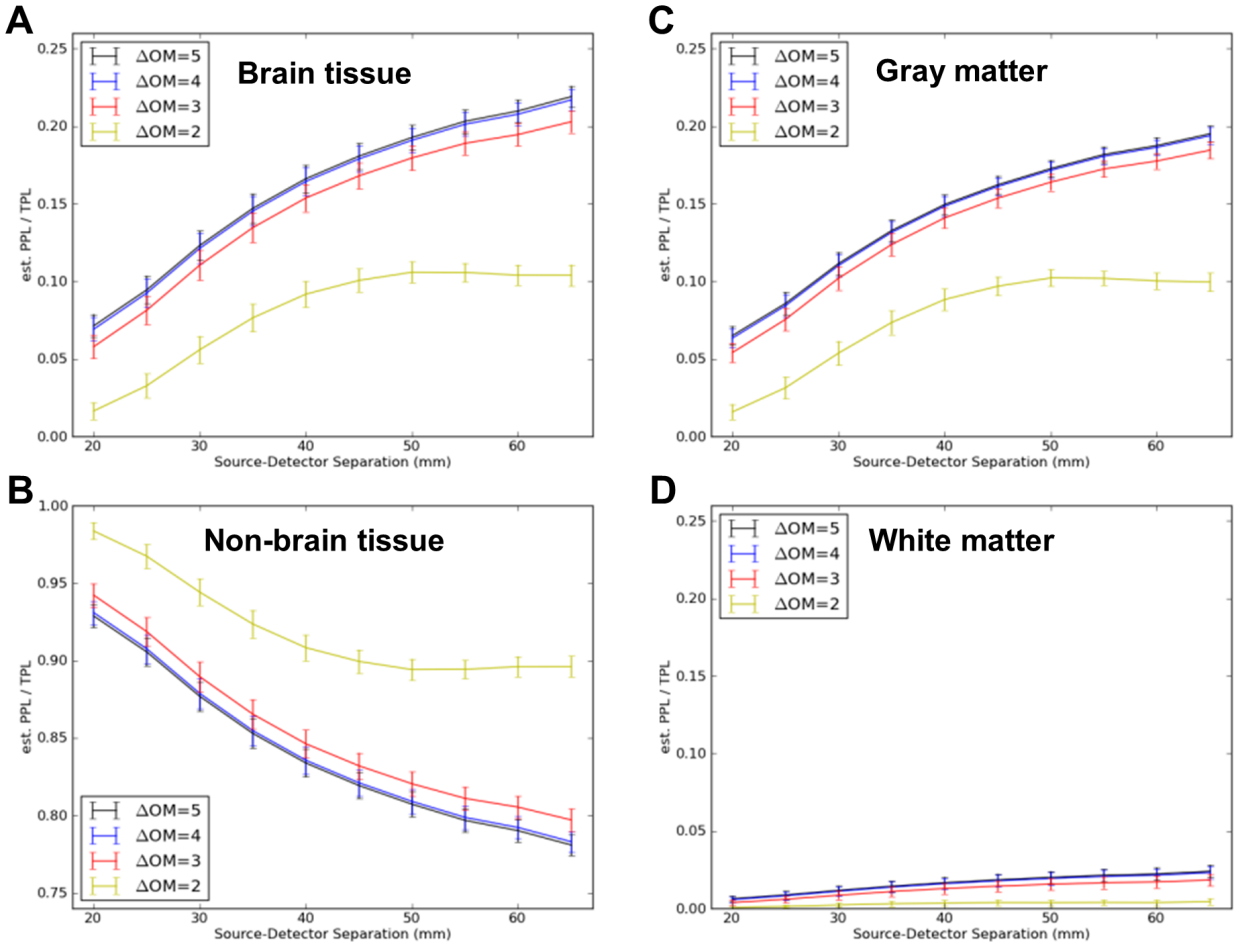
Mean proportion of total sensitivity to the tissue types indicated as a function of source-detector separation. Errorbars represent standard errors across all nineteen locations in the International 10–20 System. Separate curves represent pre-thresholding of the sensitivity (3-point Green's function) maps at 5, 4, 3, or 2 orders of magnitude (OM) reduction in sensitivity compared to peak, representing progressively less optimal NIRS measurement systems. (A) Sensitivity to brain tissue = gray plus white matter. (B) Non-brain tissue = CSF plus skull plus scalp. (C) Gray matter only. (D) White matter only.

**Figure 5:**
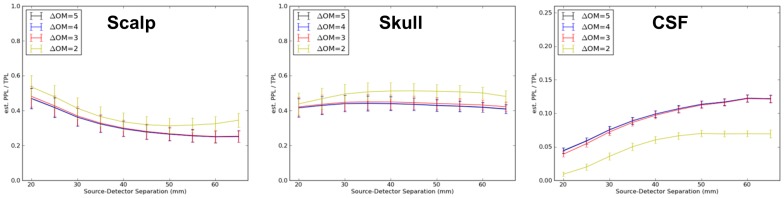
Mean proportion of total sensitivity to scalp, skull, and CSF as a function of source-detector separation. Errorbars represent standard errors across all nineteen locations in the International 10–20 System. Separate curves again represent pre-thresholding of the sensitivity (3-point Green's function) maps at 5, 4, 3 or 2 orders of magnitude (OM) reduction in sensitivity compared to peak.

Figure 4 shows quantitatively the average proportion of sensitivity to brain tissue (gray+white matter) and non-brain tissue (scalp+skull+CSF) as a function of SD separation, along with separate estimates for gray and white matter. The fluence mean and variability within the indicated tissue type(s) was computed across the nineteen 10–20 locations and error bars hence represent an estimate of the variability over these 19 head locations. The results were plotted as a function of SD separation. Two main points should be noted. First, there was notable sensitivity to brain tissue at a separation of 20 mm, representing approximately 6% of the total sensitivity profile. (Smaller separations are not included because there were too few source-detector pairs having a midpoint of measurement within 10 mm of the 10–20 positions for reliable estimation.) Second, at the largest SD separation (65 mm), the estimated sensitivity to brain tissue reached approximately 22% of the total sensitivity profile Thus, even at these larger-than-typical separations, the NIRS measurement is substantially more sensitive to the scalp, skull and CSF tissue compartments than the gray and white matter components. Figure 5 provides equivalent curves for scalp, skull and CSF tissues.

The sensitivity to gray matter, of particular interest for NIN measurements, was largely linear up through 45 mm separations, at which point diminishing returns were observed for larger separations. Linear regression across the 20 through 45 mm separation values for gray matter revealed a slope of 0.04±0.003 per centimeter. That is, for every 10 mm increase in SD separation, sensitivity to gray matter increased an additional 4% (from 6% at 20 mm, to 16% at 45 mm).

### Sensitivity as a Function of Instrument Performance

The entire analysis described above was repeated after masking the 3-point functions at 5 or (separately) 4, 3 or 2 orders of magnitude sensitivity loss from peak (separate curves in each panel in Figure 4 and Figure 5). The results with no masking and masking at 5 orders of magnitude were indistinguishable and hence only the latter is plotted for clarity. The analyses at 3 and 4 orders of magnitude, simulating progressively lower performing NIRS instrumentation, provided qualitatively similar curves, although absolute sensitivity was reduced (downward shifts) to both gray and white matter layers as progressively more of the 3-point function was masked. The sensitivity decrement in gray matter was just significant (p<0.05) at most separations when masking after 3 orders of magnitude, and reflected an 8–10% decrease in sensitivity. The magnitude of this decrement was roughly equivalent to the decrement associated with a 3 mm increase in SD separation. Masking after only 2 orders of magnitude sensitivity change – approximately equivalent to an instrument with 40 dB dynamic range – exhibited substantial and significant reductions in sensitivity, up to 50%, in all intracranial tissue classes (CSF, gray and white matter).

### NIRS Depth Sensitivity in the Brain

**Figure 6:**
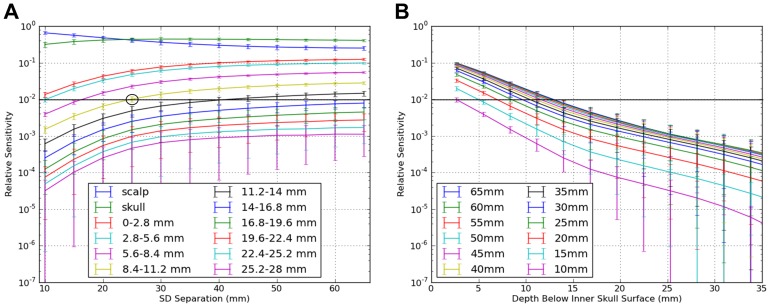
Mean NIRS depth sensitivity in the brain plotted in two orthogonal ways, by SD separation. (A) The top two traces represent scalp (blue) and skull (green) sensitivity. Sensitivity to scalp and skull were equal at a SD separation of 25 mm. On average, 1% or more of the sensitivity profile was achieved for all of the most superficial 11.2 mm of the intracranial volume at SD separations of 25 mm or greater (circle). (B) Intracranial sensitivity in depth as a function of source-detector separation (excluding scalp and skull). At all separations, sensitivity decreases exponentially with depth (i.e., linear curves through ∼15 mm depth on this semilog plot).

To estimate the NIRS sensitivity to intracranial tissue as a function of depth in the Colin27 model we used the intracranial shells depicted in Figure 1C and the described partitioning method. Figure 6 plots the average sensitivity to each shell – starting with scalp, then skull, then each successive ∼2.8-mm intracranial shell – averaged over all 10–20 positions. Note that the first such shell (0–2.8 mm) typically intersected a substantial portion of CSF plus a modest amount of gray matter. The next intracranial shell (∼2.8–5.6 mm below the inner skull surface) typically contained substantially less CSF and considerably more gray matter. The third intracranial shell (5.6–8.4 mm deep) begins to have more contact with white matter voxels, and so on towards the center of the brain (cf. Figure 1C).

In Figure 6, two primary effects are prominent. First, as SD separation increases, the sensitivity to all layers except scalp also increases (Figure 6A). For example, at a 20 mm separation, scalp and skull provided approximately 84% and 15% of the NIRS measurement sensitivity, whereas at 55 mm SD separations they provided closer to 35% and 45% of the NIRS sensitivity. The arbitrarily-selected 1% sensitivity line (y-axis = 10^−2^) was exceeded for approximately the outermost 11.2 mm of intracranial volume, starting at a 25 mm SD separation.

The 3-point Greens function (Eqn. 1) is essentially exponential, and hence we plotted log_10_(sensitivity) as a function of depth into the intracranial space (Figure 6B). At any given SD separation, sensitivity was indeed observed to decrease exponentially with depth. Non-linear regression analysis was performed on the sensitivity data (from the nineteen 10–20 positions) using an exponential formula of the following form:
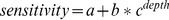
(6)


**Figure 7:**
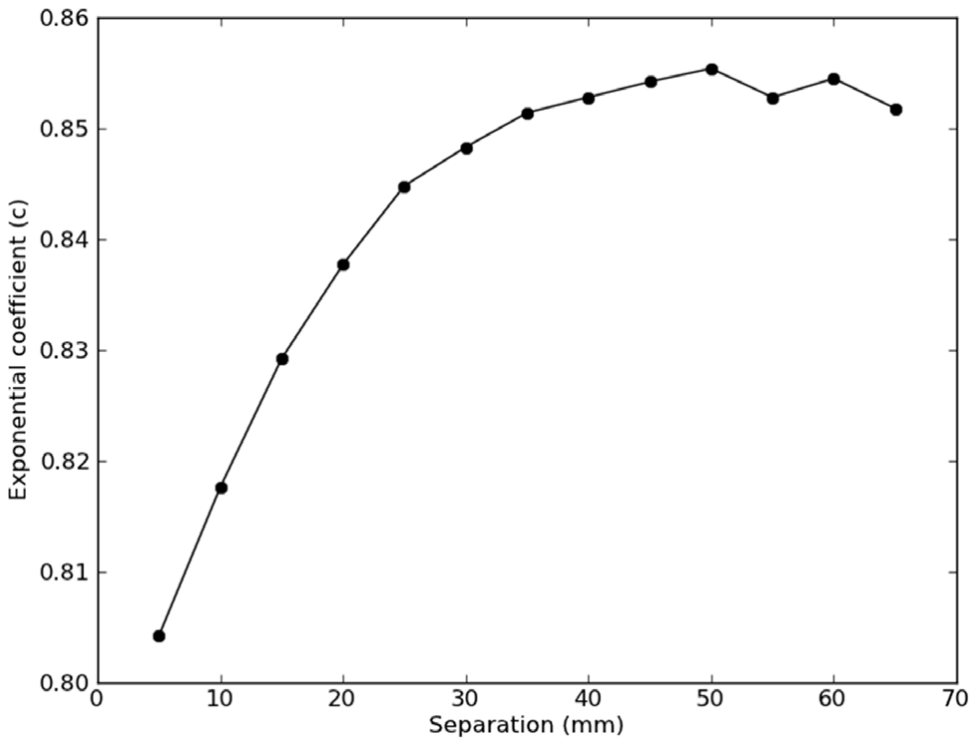
Fitted exponential decay coefficient, c, from the sensitivity function in Eqn. (6) as a function of SD separation. The asymptote at ∼40 mm separations means that further increasing the SD separation provides diminishing returns for NIRS sensitivity to brain function.

The regressions revealed significant b and c coefficients at each separation, with models accounting for 53%–91% of the total variance (see Table 2). As expected, the sensitivity at the innermost edge of the skull increased significantly as SD separation increased (T(11)  = 16.2, p<0.0001; slope = ; adjusted R^2^ = 0.96). In addition, the exponential (decay) coefficient c increased significantly with larger SD separations, reflecting a slower loss in sensitivity with depth at larger SD separations. However, the c coefficient plateaued at approximately a 40 mm separation (Figure 7).

**Table 2:** Regression results for sensitivity as a function of depth.

SD	Model	Coef.	Std.			95% Conf. Interval		N	Sensitivity at Depth (mm)		
Sep.	Param	Value	Err.	T	p	Low	High	Adj. R2	0	5	10
**5**	a	0.0000	0.0001	−0.6	0.550	−0.0002	0.0001	378			
	b	0.0058	0.0003	19.3	0.000	0.0052	0.0064	0.526	0.58%	0.19%	0.06%
	c	0.8042	0.0185	43.4	0.000	0.7678	0.8406				
**10**	a	−0.0001	0.0001	−0.7	0.491	−0.0004	0.0002	399			
	b	0.0133	0.0005	25.3	0.000	0.0123	0.0143	0.647	1.32%	0.48%	0.17%
	c	0.8176	0.0132	62.1	0.000	0.7917	0.8435				
**15**	a	−0.0002	0.0003	−0.7	0.458	−0.0007	0.0003	399			
	b	0.0261	0.0009	27.8	0.000	0.0243	0.0280	0.687	2.59%	1.00%	0.38%
	c	0.8292	0.0113	73.6	0.000	0.8071	0.8514				
**20**	a	−0.0003	0.0004	−0.9	0.392	−0.0011	0.0004	399			
	b	0.0431	0.0013	32.7	0.000	0.0405	0.0457	0.753	4.27%	1.74%	0.70%
	c	0.8377	0.0091	91.8	0.000	0.8198	0.8557				
**25**	a	−0.0005	0.0005	−1.0	0.341	−0.0015	0.0005	399			
	b	0.0614	0.0017	36.5	0.000	0.0581	0.0647	0.793	6.09%	2.59%	1.09%
	c	0.8448	0.0078	107.8	0.000	0.8294	0.8602				
**30**	a	−0.0006	0.0006	−1.1	0.276	−0.0018	0.0005	399			
	b	0.0781	0.0019	41.6	0.000	0.0744	0.0818	0.832	7.74%	3.37%	1.44%
	c	0.8483	0.0067	125.7	0.000	0.8350	0.8616				
**35**	a	−0.0008	0.0006	−1.2	0.221	−0.0020	0.0005	399			
	b	0.0913	0.0020	45.8	0.000	0.0873	0.0952	0.857	9.05%	4.01%	1.75%
	c	0.8514	0.0060	141.5	0.000	0.8395	0.8632				
**40**	a	−0.0009	0.0006	−1.3	0.182	−0.0021	0.0004	399			
	b	0.1018	0.0021	49.3	0.000	0.0977	0.1059	0.875	10.09%	4.51%	1.99%
	c	0.8528	0.0055	154.1	0.000	0.8419	0.8637				
**45**	a	−0.0009	0.0007	−1.4	0.165	−0.0022	0.0004	399			
	b	0.1095	0.0021	51.5	0.000	0.1054	0.1137	0.883	10.86%	4.89%	2.17%
	c	0.8542	0.0053	162.3	0.000	0.8438	0.8645				
**50**	a	−0.0010	0.0007	−1.4	0.163	−0.0023	0.0004	399			
	b	0.1157	0.0022	52.7	0.000	0.1114	0.1200	0.889	11.47%	5.20%	2.33%
	c	0.8554	0.0051	167.9	0.000	0.8454	0.8655				
**55**	a	−0.0010	0.0007	−1.5	0.134	−0.0024	0.0003	378			
	b	0.1231	0.0022	54.8	0.000	0.1187	0.1276	0.901	12.21%	5.45%	2.40%
	c	0.8528	0.0050	171.2	0.000	0.8430	0.8626				
**60**	a	−0.0011	0.0007	−1.5	0.132	−0.0025	0.0003	378			
	b	0.1264	0.0023	56.0	0.000	0.1219	0.1308	0.905	12.53%	5.65%	2.52%
	c	0.8545	0.0048	176.9	0.000	0.8450	0.8640				
**65**	a	−0.0011	0.0007	−1.6	0.112	−0.0025	0.0003	357			
	b	0.1321	0.0023	57.6	0.000	0.1275	0.1366	0.914	13.09%	5.81%	2.54%
	c	0.8518	0.0048	178.6	0.000	0.8425	0.8612				

While depth sensitivity for a given SD separation can be estimated directly from the coefficients in Table 2 plus Eqn. (6), we also computed a “rule of thumb” formula. Averaging over the coefficients for typically used NIRS separations of 20–40 mm results in the following equation for the sensitivity in depth (S):
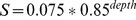
(7)


Thus, at a “typical” separation, our MCs estimate a 0.075*0.85^0^ = 0.075, or 7.5% sensitivity at the average inner surface of the skull. At a depth 5 mm into the intracranial space, the sensitivity would decrease, to 0.075*0.85^5^ = 0.033 or 3.3%. Such estimates are coarse, and assume overlying scalp and skull layers comparable to those in our Colin27 template. However, Equation (7) can provide a reasonable estimate of NIN sensitivity as a function of depth. For comparison, quantitative depth sensitivity measures at each 10–20 location for a typical SD separation of 30 mm are listed in Table 3.

**Table 3:** Estimated relative NIRS sensitivity (proportions) as a function of depth for a SD separation of 30 mm.

				Shell Depth Range (mm)						
Location	N	Scalp	Skull	0–2.8	2.8–5.6	5.6–8.4	8.4–11.2	11.2–14	14–16.8	16.8–19.6
**Fp1**	45	0.16	0.70	0.059	0.046	0.0202	0.0078	0.00272	0.00079	0.00020
		(0.03)	(0.02)	(0.009)	(0.007)	(0.0032)	(0.0013)	(0.00051)	(0.00017)	(0.00006)
**Fp2**	47	0.21	0.64	0.064	0.047	0.0190	0.0069	0.00241	0.00073	0.00019
		(0.04)	(0.03)	(0.011)	(0.008)	(0.0039)	(0.0016)	(0.00062)	(0.00022)	(0.00008)
**Fz**	66	0.26	0.52	0.047	0.046	0.0406	0.0302	0.02048	0.01339	0.00762
		(0.02)	(0.02)	(0.004)	(0.004)	(0.0032)	(0.0033)	(0.00307)	(0.00255)	(0.00187)
**F3**	78	0.15	0.64	0.084	0.071	0.0341	0.0145	0.00591	0.00217	0.00071
		(0.01)	(0.01)	(0.010)	(0.007)	(0.0030)	(0.0016)	(0.00080)	(0.00035)	(0.00013)
**F4**	91	0.17	0.64	0.085	0.066	0.0273	0.0097	0.00341	0.00111	0.00032
		(0.01)	(0.01)	(0.008)	(0.006)	(0.0024)	(0.0010)	(0.00046)	(0.00019)	(0.00007)
**F7**	76	0.65	0.21	0.055	0.046	0.0218	0.0088	0.00318	0.00111	0.00040
		(0.06)	(0.03)	(0.012)	(0.009)	(0.0041)	(0.0016)	(0.00062)	(0.00025)	(0.00012)
**F8**	71	0.69	0.20	0.041	0.036	0.0185	0.0077	0.00291	0.00114	0.00048
		(0.05)	(0.03)	(0.008)	(0.007)	(0.0033)	(0.0015)	(0.00065)	(0.00029)	(0.00015)
**C3**	90	0.34	0.41	0.068	0.069	0.0520	0.0297	0.01501	0.00713	0.00325
		(0.02)	(0.01)	(0.005)	(0.005)	(0.0044)	(0.0029)	(0.00169)	(0.00087)	(0.00043)
**C4**	83	0.36	0.46	0.052	0.053	0.0389	0.0220	0.01047	0.00467	0.00201
		(0.02)	(0.01)	(0.005)	(0.005)	(0.0037)	(0.0023)	(0.00126)	(0.00064)	(0.00031)
**Cz**	43	0.41	0.30	0.031	0.034	0.0350	0.0358	0.03275	0.02818	0.02398
		(0.02)	(0.02)	(0.002)	(0.002)	(0.0016)	(0.0014)	(0.00168)	(0.00159)	(0.00132)
**P3**	66	0.26	0.52	0.087	0.073	0.0359	0.0146	0.00566	0.00210	0.00070
		(0.02)	(0.01)	(0.009)	(0.007)	(0.0044)	(0.0023)	(0.00107)	(0.00047)	(0.00019)
**P4**	65	0.25	0.49	0.095	0.081	0.0452	0.0209	0.00901	0.00346	0.00118
		(0.02)	(0.01)	(0.007)	(0.005)	(0.0038)	(0.0024)	(0.00129)	(0.00061)	(0.00025)
**Pz**	38	0.48	0.34	0.037	0.037	0.0337	0.0253	0.01693	0.01081	0.00683
		(0.06)	(0.03)	(0.007)	(0.007)	(0.0064)	(0.0051)	(0.00369)	(0.00245)	(0.00159)
**O1**	34	0.17	0.56	0.132	0.093	0.0333	0.0103	0.00303	0.00084	0.00021
		(0.01)	(0.01)	(0.007)	(0.005)	(0.0027)	(0.0011)	(0.00039)	(0.00013)	(0.00004)
**O2**	43	0.19	0.51	0.133	0.096	0.0400	0.0147	0.00528	0.00192	0.00065
		(0.01)	(0.02)	(0.009)	(0.005)	(0.0032)	(0.0020)	(0.00111)	(0.00056)	(0.00024)
**T3**	80	0.68	0.12	0.083	0.066	0.0304	0.0112	0.00387	0.00127	0.00038
		(0.02)	(0.01)	(0.008)	(0.006)	(0.0029)	(0.0011)	(0.00043)	(0.00016)	(0.00006)
**T4**	66	0.70	0.13	0.076	0.057	0.0264	0.0103	0.00371	0.00123	0.00037
		(0.03)	(0.01)	(0.009)	(0.006)	(0.0030)	(0.0012)	(0.00046)	(0.00016)	(0.00006)
**T5**	62	0.35	0.49	0.078	0.052	0.0169	0.0048	0.00134	0.00035	0.00006
		(0.03)	(0.02)	(0.012)	(0.008)	(0.0030)	(0.0010)	(0.00034)	(0.00013)	(0.00004)
**T6**	63	0.40	0.47	0.074	0.043	0.0131	0.0036	0.00094	0.00021	0.00002
		(0.05)	(0.03)	(0.011)	(0.007)	(0.0025)	(0.0008)	(0.00024)	(0.00007)	(0.00002)

## Discussion

Utilizing 3,555 Monte Carlo simulations, we generated quantitative estimates of the spatial sensitivity and associated variability of non-invasive NIRS to brain tissue in the Colin27 brain. Sensitivity as a function of source-detector separation was qualitatively consistent with previous reports [23], [24], [31], [32], [36]. Global sensitivity to brain tissue reached 20% (and gray matter = 17%) of the total sensitivity at a SD separation of 55 mm, with the remaining sensitivity coming from scalp, skull and CSF. More typical SD separations of 25–35 mm exhibited brain sensitivities in the range of 8–13%. As expected, sensitivity was degraded when we simulated under-performing NIRS instrumentation, with a typical sensitivity reduction equivalent to the decrease expected from a 2.5 mm increase in SD separation. Performance appeared to drop quickly with more severely degraded instruments (as dynamic range fell from ∼60 dB to ∼40 dB). Finally, the sensitivity in depth through the intracranial space was found to be exponentially decreasing, with detailed exponential coefficients given in Table 2, and an approximation for sensitivity-in-depth given by Eqn. (7). NIN signals thus appear strongly biased to absorption changes occurring in the outer 10–15 mm of the intracranial space. While these results were based on the thinned-scalp version of Colin27, we also performed a full set (3,555) of MC simulations on the original, thicker scalp. Qualitatively, we replicated all results reported herein, with the primary difference being uniformly but modestly lower sensitivity to brain tissue in the thicker-scalp model.

### Sensitivity versus SD Separation

While an increase in brain sensitivity was anticipated with larger SD separations [31], [32], [35], [40], the magnitude and variability of this relationship around the head, and in a realistic geometry, were not well characterized. Our MC results suggest that, globally, as much as 6% of the NIRS sensitivity comes from brain tissue at SD separations of 20 mm. This probability rose sigmoidally over the range of separations we investigated, increasing to 20% of the NIRS sensitivity coming from brain tissue at a 55 mm separation. This is qualitatively consistent with recent studies that examined only small regions in different head models [35], [39], [40].

Our results did quantitatively differ to some degree from this prior work. For example, our observed sensitivity of ∼10% at a separation of 30–35 mm was slightly higher than that found when using the Chinese head dataset [55], for which 8% was reported at the same 30–35 mm separation [39]. However, that study – as well as the others that considered realistic head models – only examined very small portions of a different head model, whereas our mean and variability estimates apply to the entire Colin27 head.

Variability in NIRS sensitivity to brain had not been previously assessed. In our study, variability in sensitivity was modest within regions, ranging from 10–18%. Variability was somewhat more substantial between regions, with standard errors across the 10–20 locations spanning 20% of the mean sensitivity values. The variability around the head, therefore, appears to be an important contributor to NIN sensitivity. This variability is presumed to be related to the different tissue layering found across different head regions.

There was a notable gain in sensitivity with SD separation, particularly at greater than 25 mm separations. In one prior study, it was recommended that NIRS researchers avoid SD separations greater than 30–35 mm [39]. While this does not appear to be supported by our findings, it is important to note that our estimates do not consider the achievable signal-to-noise ratio (SNR). If an instrument cannot detect a reliable signal at 40 or 50 mm, larger separations will not improve brain sensitivity. A trade-off therefore needs to be made between greater sensitivity to deeper layers and the resulting loss in SNR. All NIRS instruments have a limit on the separation where reliable SNR can be achieved, which is readily determined by experimentation. We investigated a broad range of separations, spanning the typical capabilities of current instruments, to help individual investigators determine this trade-off for specific devices.

Based on our results, therefore, NIRS probes for non-invasive neuromonitoring in adults should ideally be designed with 30–35 mm SD separations, or larger, assuming the instrument can provide adequate SNR at those separations. Note that this recommendation should also be weighed against spatial resolution when choosing SD separations. As SD separations increase, the effective resolution of the NIRS measurement decreases. In some studies, the need for a higher spatial resolution (and hence shorter SD separations) may outweigh the need for more or deeper sensitivity (and hence larger SD separations).

For close SD pairs, separations of 20 mm included on average 6% of the photon fluence (sensitivity) attributable to brain tissue. Thus, the commonly used rule of thumb – namely, that measurements with 20 mm or smaller separations are minimally sensitive to the brain in adult humans may need to be revised. Clearly, brain activation that is both strong and superficial could still be detected with a 20 mm separation. As mentioned earlier, this would be even more prominent if superficial layers exhibit little or no change in optical properties, or such changes are eliminated or suitably accounted for. Since there is substantial vascularization of and hemodynamic activity in scalp, skull and pial vessels, experimental manipulations to eliminate such signals may be difficult. When seeking to independently measure peripheral tissues like scalp and skull, as when making multi-distance measurements to correct for superficial layer hemodynamics [56], [57], [58], [59], [60], SD separations shorter than 10 mm should be strongly considered.

### Sensitivity and NIRS Instrument Performance

When comparing the different curves within panels in Figure 4 and Figure 5, the peak sensitivity dropped only about 10% when assuming a NIRS instrument with a lower dynamic range or optimization: for gray matter at a separation of 55 mm, the reduction was from 0.20 to 0.18. This dropped more precipitously (by up to 50% of the original value) to 0.10 with poor dynamic range or optimization. This suggests the importance of optimizing instrument sensitivity and reducing all types of noise and signal interference when making NIN measurements [57], [60], [61]. This appears to be particularly for instruments with inherently modest dynamic range (<60 dB).

### Brain Sensitivity in Depth

Our shell-based depth analysis revealed exponentially decreasing sensitivity with depth at all SD spacings, with expectedly different intercepts at each separation. Upon reaching 11.2 mm into the brain, relative sensitivity greater than 1% was only found with 25 mm or greater separations. Thus, in our Colin27 head model, substantial separation appears required to achieve even modest sensitivity past the outer 10 mm of intracranial space. Two important qualifiers should be noted. First, the sensitivity beyond 10 mm into the intracranial space is strongly limited by the thickness of the overlying tissue layers, including scalp and skull. In our model these layers together averaged ∼13 mm thick. Other individuals with thicker or thinner scalp and skull layers would be expected to have decreased or increased overall sensitivities, respectively. While these are not controllable parameters, they should be considered with respect to study design. Second, the ability to detect absorption changes is also a function of contrast and noise. A large enough change in optical properties (i.e., absorption or scattering), a large enough region of change, or a low enough noise floor (including noise generated from systemic physiological processes as discussed above), could enable detection of events occurring substantially deeper than 10 mm. Mathematically, the detectability of a specific functional event depends on the contrast-to-noise ratio. Usually one uses CNR  = 1 (contrast is equal to the noise floor) to define the minimum detectable contrast. Hence, we have the equation 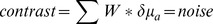. We discussed at length the role of 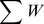 in this paper. However, 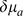 (the change in optical properties) is clearly equally important, such that a doubling of absorption coefficients would lead to a doubling of the detected contrast.

In terms of noise, if there was precisely zero change in optical properties in overlying layers (e.g., scalp and skull), or the changes in overlying layers can be otherwise accounted for, then any detected change must have come from deeper layers, regardless of the *relative* sensitivity at that depth. The magnitude of optical contrast or the level of noise suppression required for detection of brain activity at, say, a 20 mm depth into the intracranial space remains to be investigated.

### Study Limitations

Hair is invisible to MRI, and hence was not considered in this study. Hair can mechanically interfere with NIN measurements and also has its own absorption spectrum, providing two separate reductions in NIRS sensitivity. However, in the absence of motion, hair is expected to be a substantially less dynamic absorber than scalp or skull tissue, as it lacks hemodynamic and most other time-dependent physiologic processes. Thus, while the sensitivity in regions with hair will generally be lower than in regions without hair (all else being equal), the reductions are expected to be essentially fixed for a given hair color and density. To our knowledge, no quantification of the effect of hair on NIN measurements has yet been performed. Also, the specific methods used to segment the Colin template may have led to over- or under-estimates of tissue thicknesses or volumes. Proper assessment of this hypothesis requires re-segmentation and re-running all Monte Carlo simulations and follow-on analyses and hence was beyond the scope of this study. Within the segmented regions, our MC simulations used average optical properties, largely to reduce the number of separate MCs that had to be performed. While we have performed a few MC runs using wavelength-specific optical properties and only observed subtle differences in sensitivity profiles, systematic quantification or comparison across wavelengths in realistic head geometries remains as future work. Finally, while our MC simulations covered the Colin27 head model comprehensively, we only examined a single head model. Different individuals will exhibit different scalp and skull thicknesses, CSF distributions, cortical folding patterns, white matter composition, and so forth. For example, females have been found to have different skull thickness distributions than males [62], [63], children have overall thinner scalps and skulls than our model [64], older individuals may have different baseline scattering properties [65], and individuals vary in terms of the amount and distribution of CSF [66]. Direct assessments of each of these parameters on NIN sensitivity remain to be performed. However, our large set of MC simulations did encompass a substantial range of scalp, skull and CSF combinations, and our associated variability estimates provide a guide to the importance of some of these issues.

## Conclusions

This study represents the most comprehensive characterization conducted to date of NIRS sensitivity to brain tissue, including the importance of source-detector separations, variability in NIRS sensitivity around the head, the influence of NIRS instrument performance, as well as NIN sensitivity in depth. Our results suggest that increasing the source-detector separation past 20 mm monotonically increases sensitivity to brain tissue, and hence the larger the separation the better in terms of brain sensitivity. However, diminishing returns appear to begin around 40–50 mm SD separations, and sensitivity must also be balanced against the SNR that can be achieved with any particular instrument at large separations, as well as the spatial resolution required. Our MC simulations further suggest that, while the depth sensitivity of NIRS is not strictly limited, NIRS sensitivity decreases exponentially with depth into the intracranial space and hence NIN signals are strongly biased towards the outermost 10–15 mm of the intracranial volume. The detailed quantitative information provided here can help investigators better design and plan experiments, head probes and instruments for making NIRS measurements, as well as providing guidance when interpreting NIRS studies in terms of the likely sources of the observed signals.
